# Brooding Temperature Alters Yolk Sac Absorption and Affected Ovarian Development in Goslings

**DOI:** 10.3390/ani12121513

**Published:** 2022-06-10

**Authors:** Zhengquan Liu, Xingyong Chen, Yutong Zhao, Jingzhou Peng, Daoyou Chen, Shiqi Yu, Zhaoyu Geng

**Affiliations:** 1 College of Animal Science and Technology, Anhui Agricultural University, No. 130 Changjiang West Road, Hefei 230036, China; zliu13155@gmail.com (Z.L.); z18255494174@163.com (Y.Z.); pjz1113494031@163.com (J.P.); 15856307233@163.com (D.C.); yu1845674423@163.com (S.Y.); gzy@ahau.edu.cn (Z.G.); 2 Anhui Province Key Laboratory of Local Livestock and Poultry Genetic Resource Conservation and Bio-Breeding, Anhui Agricultural University, No. 130 Changjiang West Road, Hefei 230036, China

**Keywords:** brooding temperature, gosling, ovary, yolk sac

## Abstract

**Simple Summary:**

It is well known that brooding temperature affects yolk sac absorption so as to affect the growth and development of geese. In order to explore the brooding temperature on the absorption of yolk sac and the ovary development of goslings, the weight and fatty acid composition of yolk sac, body weight, ovary weight and structure was measured. The expression of ovary development related genes was quantified. The results suggested that 29 °C brooding temperature in the early stage could promote the absorption of fatty acids from yolk sac, which is conducive to ovary and body weight, and the expression of ovarian development-related genes of *CHK1*, *FGF12*, and *SMAD4*. The results could provide guidance on gosling brooding for farmers.

**Abstract:**

In order to explore the brooding temperature on the absorption of yolk sac and the ovary development of goslings, 126 1-day-old female goslings were randomly divided into three groups with three replicates in each group. The brooding temperatures were set at 32 °C, 29 °C and 26 °C (represent G32, G29 and G26), respectively, in each group. At 48, 60 and 72 h, two goslings from each replicate were weighed, and the yolk sac was collected and weighed. The fatty acid composition of yolk sac fluid was determined by gas chromatography-mass spectrometry (GC-MS). At 1, 2, 3, and 4 weeks of age, goslings from each replicate were weighed, the ovaries were weighed and fixed for hematoxylin-eosin (HE) staining, Cell cycle checkpoint kinase 1 (*CHK1*), fibroblast growth factor 12 (*FGF12*) and Sma-and Mad-related protein 4 (*SMAD4*) which related to regulation of ovarian development were determined by qRT-PCR. The body weight of G29 and G26 was significantly higher than that of G32 at 72 h (*p* < 0.05). The contents of C14:0, C16:0, C18:2n6c and total fatty acid (ΣTFA) from G32 were significantly higher than that of G26 (*p* < 0.05), and the contents of C18:1n9t and C22:0 in G29 were significantly higher than that of G26 (*p* < 0.05). The ovary index, ovary and body weight were significantly higher in G29 than those of G32 and G26 at 2 weeks of age (*p* < 0.05). The number of primordial follicles, number of primary follicles and diameter of primary follicles were significantly higher in G29 than those in G32 and G26 at 4 weeks of age (*p* < 0.05). In G29, the expression of *CHK1* and *SMAD4* was significantly higher than that in G32, and the expression of *FGF12* and *SMAD4* was significantly higher (*p* < 0.05) than that in G26 at 2 and 4 weeks of age. In conclusion, brooding temperature at 29 °C could promote the absorption of fatty acids in yolk sac, body weight gain, and ovarian development through up-regulating the expression of *CHK1*, *FGF12* and *SMAD4*.

## 1. Introduction

Successful brooding directly determines the survival rate, growth and development of goslings and the egg production rate of adult geese [1]. It is well known that brooding temperature affects yolk sac absorption so as to affect the health of birds. High ambient temperatures of 35 °C reduced food intake and body weight in chicks, and increased diencephalic oxidative damage 48 h posthatch [2]. Low brooding temperature around 20 °C during the first seven days post-hatch stressfully decreased broiler bone development and reduced chicks’ body weight [3], whereas the weight of yolk sac and bursa of Fabricius was not affected. The food intake and body weight gain of ducklings at 36 °C were lower than those at 22 °C [4]. However, studies suggested that ducklings at 20 °C significantly showed reduced growth performance, increased feed intake, and reduced average body weight gain and feed conversion [5]. Geese are cold resistant poultry and farmers would like low temperature brooding to save heating energy. There are still no reports about whether low temperature brooding could affect yolk sac absorption and further development in geese.

In newborn birds, the yolk sac, which is absorbed into the abdominal cavity, becomes an important organ in the transition from endogenous nutrient absorption to exogenous feed digestion [6]. The yolk sac is connected to the small intestine through the yolk sac pedicle. The yolk maintains the growth and development of birds in the early post-hatch period until the gut could stably utilize exogenous feed. Proper utilization of adequate amounts of nutrients (glycogen, glucose, fatty acids, and peptides) stored in the yolk sac is a key factor for the growth and development of chicks before exogenous diet provision [7], and the ovarian development of newborn chicks also mainly depends on the absorption of nutrients from the yolk sac [8]. It is clear that the yolk sac could supply the nutritional needs of the chicks within the first 3 days. Therefore, inadequate absorption of the yolk sac could lead to decreased ovarian germ cell proliferation in early goslings and reduced egg-laying performance in later stages. 

The ovary is an important reproductive organ of poultry, and the activation and development of primordial follicles during the early brooding period plays a key role in the later egg laying of birds. Studies by Rozenboim et al. [9] suggested that short and long heat exposure caused significant hyperthermia and reduction of egg production, egg weight, ovarian weight, and the number of large follicles. It could be seen that the brooding temperature is highly associated with ovarian development in birds. Cell cycle checkpoint kinase 1 (*CHK1*) mediated phosphorylation of *CDH1* so as to promote follicle granulosa cell proliferation [10]. Overexpression of fibroblast growth factor 12 (*FGF12*) increased granulosa cell proliferation, while *FGF12* inhibition promoted granulosa cell apoptosis and induced follicle atresia [11]. The expression of Sma-and Mad-related protein 4 (*SMAD4*) in pre-hierarchical follicles could promote its development into hierarchical stage [12]. Therefore, the ovarian development could be indirectly reflected by the expression level of these granulosa cell proliferation related genes in birds. 

In this study, yolk sac absorption, body weight and ovary development were compared in geese under different brooding temperatures. The results could provide guidance on gosling brooding for farmers.

## 2. Materials and Methods

### 2.1. Animals and Experimental Materials

**Figure 1:**
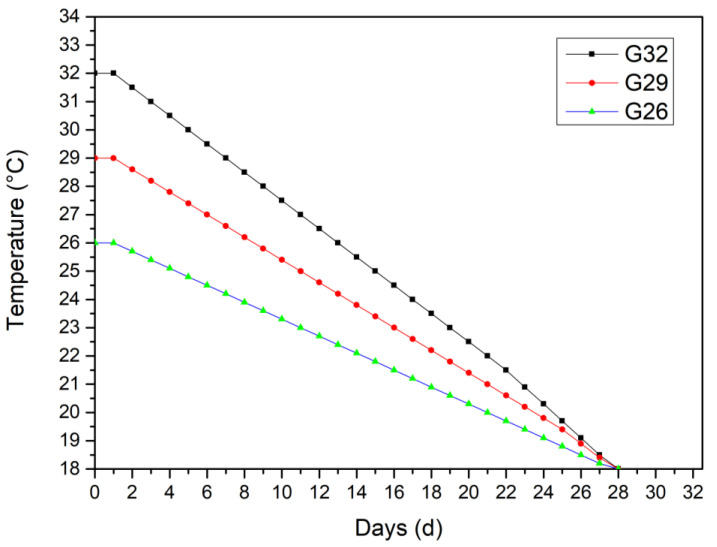
Temperature regime for gosling brooding at the first 4 weeks of age.

A total of 126 1-day-old female goslings (Minzhiyuan Geese Industry Co., Ltd., Dingyuan, China), with similar body weightd of 92.62 ± 2 g, were randomly divided into three groups (*n* = 126) with three replicates in each group and 42 goslings in each replicate. Goslings were raised in 32 °C, 29 °C, and 26 °C, respectively, for each group, namely G32, G29 and G26 during the first 72 h in an environmental controlled room with continuous fluorescent illumination. The room temperature was decreased to 18 °C at 4 weeks of age in all the three groups (Figure 1). All goslings were placed on wire floor, and each separated unit served as one replicate. Water and feed in pellet form were supplied ad libitum and diet nutrition met the NRC recommendations [13]. At 48, 60, and 72 h post-hatch, two goslings from each replicate were weighed and then slaughtered for yolk sac collection. The yolk sac was weighed and stored in −20 °C for fatty acid composition and content determination. At 1, 2, 3, and 4 weeks of age, two goslings from each replicate were weighed and then slaughtered for the ovary collection. The ovary was weighed, and one part was stored in 4% paraformaldehyde fixative, the left was stored in liquid nitrogen and transferred to −80 °C for RNA extraction.

### 2.2. Determination of Fatty Acids in Yolk Sac Fluid

The composition and content of fatty acids were analyzed according to Walczak et al. [14], with appropriate modifications. A total of 1.5 g yolk sac was weighed and placed into 10 mL centrifuge tube together with 0.66 mL internal standard of C11:0 (5 mg·L^−1^). The sample was ground (JX-48, Jinxing, Shanghai, China) for 2 min at 60 Hz with 95% ethanol (0.66 mL) and of pure water (1.33 mL), and then transferred to a flask containing 33 mg of pyrogallol, 33 mg of zeolite, and 3.3 mL of hydrochloric acid (8.3 mol·L^−1^). After incubation at 75 °C for 40 min, the sample was mixed with 3 mL of 95% ethanol, 10 mL of ether and petroleum ether mixture (*V*:*V* = 1:1), and transferred to a separating funnel for standing for 5 min. The ether layer extract was collected into a flask and transferred to a rotary evaporator (RE-2002, Exceed, Shanghai, China) for evaporation until is reached a constant weight. A total of 1.6 mL of 2% NaOH-methanol solution was added to the flask and incubated in water bath at 80 °C for 3 min, and then 1.4 mL of 15% boron trifluoride methanol solution was added and incubated in a water bath at 80 °C for 3 min. The flask was cooled to room temperature and then mixed with 2 mL of n-heptane and 3 mL of saturated aqueous sodium chloride solution. After standing for 5 min, the upper layer of liquid was transferred to another tube and mixed with 0.6 g anhydrous sodium sulfate. Then the supernatant was filtered through a 0.22 μm membrane and transferred into a GC injection vial for GC-MS analysis. 

The separated fatty acid methyl ester (FAME) was applied to a DEGS capillary column (DB-WAX, 30 m × 0.25 mm × 0.25 mm, Agilent Technologies Inc., Santa Clara, CA, USA) and analyzed with a flame ionization detector by Agilent 7980N gas chromatography (Agilent Technologies, Santa Clara, CA, USA). The initial temperature of the column oven was set at 60 °C for 2 min, followed by heating at 15 °C/min to 230 °C and held for 19 min. The injector and flame ionization detector were maintained at 240 °C, and the injection volume was 1 μL at a split ratio of 10:1. The carrier gas used was nitrogen at a flow rate of 0.8 mL/min. 

Identification of FAME was performed by comparing the retention times to the FAME standards (CDAA-252795, Shanghai Anpu Experimental Technology Co., Ltd., Shanghai, China). The quantity of FAME was analyzed by using C11 as the internal standard according to the formula listed in Walczak et al. [14]. 

### 2.3. Ovarian HE Staining 

Ovarian HE staining was performed according to Guo et al. [15]. Sections were observed using an optical microscope (IX73, Olympus, Tokyo, Japan). The number and diameter of follicles were counted and measured in three randomly selected fields (1 × 1 mm) of each stroma section. Only follicles with visible oocyte nuclei were counted and measured to avoid double counting. Follicles were classified as primordial follicles and growing follicles according to the layer and shape of granulose cells. The primordial follicles are composed of an oocyte surrounded by flattened pregranulosa cells, while primary follicles contain one or more cubic granulosa cells around the oocyte [15].

### 2.4. Total RNA Extraction, cDNA Synthesis and Quantitative Real-Time PCR (qRT-PCR) Analysis

The extraction of total RNA from ovarian tissue was carried out according to the instructions of the total RNA extraction kit (10606ES60, Shanghai Yisheng Biotechnology Co., Ltd., Shanghai, China). The integrity and concentration of the extracted total RNA were detected by NanoDrop 2000 (Thermo Fisher, Waltham, MA, USA). The cDNA was synthesized by a cDNA synthesis kit (11123ES60, Shanghai Yisheng Biotechnology Co., Ltd., Shanghai, China), and the synthesized cDNA was stored at −80 °C for qRT-PCR analysis.

Fluorescence quantitative PCR primers were designed according to the sequences listed in GenBank, and glyceraldehyde-3-phosphate dehydrogenase (GAPDH) was set as the internal reference (Table 1). Primer sequences were synthesized by General Bio (Beijing, China). Fluorescence quantification was performed using a Hieff^®^ qPCR SYBR Green Master Mix kit (11202ES08, Shanghai Yisheng Biotechnology Co., Ltd.) on an ABI-7500 instrument (Thermo Fisher Company, Waltham, MA, USA). 

The reaction system was 10 μL including 5 μL Hieff^®^ qPCR SYBR Green Master Mix, 0.2 μL upstream and downstream primers, 0.6 μL cDNA, and 4 μL ddH2O. The reaction started from pre-denaturation at 95 °C for 5 min, and was followed by 40 cycles including denaturation at 95 °C for 10 s, annealing at 60 °C for 20 s, and extension at 72 °C for 20 s. Each sample was analyzed in triplicate. A melting curve was used to verify amplification specificity. The relative expression of target genes was calculated based on the 2^−ΔΔCT^ method.

### 2.5. Statistical Analysis

Data were processed using the statistical software SPSS 20.0 by one-way ANOVA. For each parameter, distribution of means and residuals was examined to verify model assumptions. In case data were not normally distributed, a log transformation was performed. The generalized linear model (GLM) was performed, and the model used for these variables was [16]:

Y = μ + T + e

where Y = dependent variable, μ = overall mean, T = temperature (32 °C, 29 °C, or 26 °C), e = standard error.

All the data were expressed as “mean ± standard error”. 

## 3. Results

### 3.1. Effect of Temperature on Yolk Sac and Body Weight at Each Stage of Gosling

The body weight in G29 and G26 was significantly higher than that in G32 at 72 h (*p* < 0.05, Table 2). Brooding temperature had no significance on body weight before 72 h (*p* > 0.05, Table 2). The weight of the yolk sac showed no significant difference among groups at each brooding stage (*p* > 0.05, Table 2).

### 3.2. Effect of Temperature on Composition and Content of Fatty Acids from Yolk Sac Fluid 

**Figure 2:**
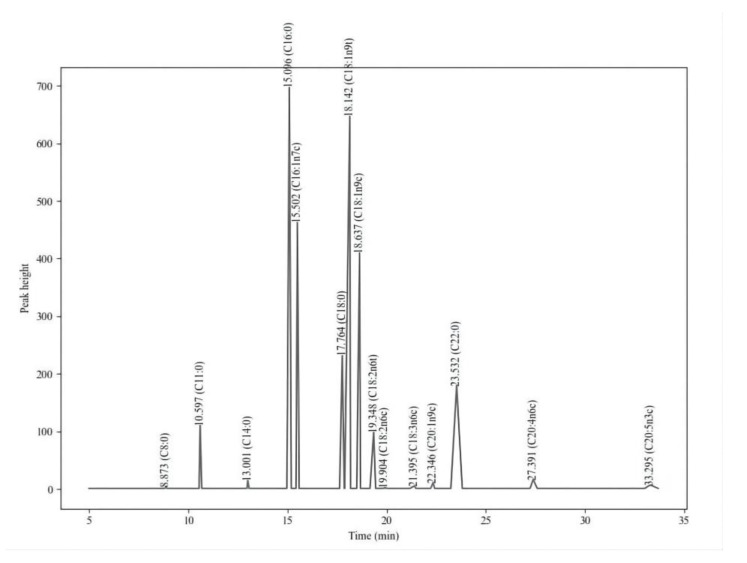
GC-MS total ion profile of a yolk sac sample.

A yolk sac consists of 14 fatty acids, including C8:0, C14:0, C16:0, C18:0, C22:0, C16:1, C18:1n9t, C18:1n9c, C18:2n6t, C18:2n6c, C20:1, C18:3n6, C20:4n6 and C20:5n3 in goslings (Figure 2). The SFA, MUFA and PUFA in the yolk sac are mainly composed of C16:0, C18:0, C22:0, C16:1, C18:1n9t and C20:4n6, which account for 32%, 5%, 11%, 18%, 19% and 3% of total fatty acids, respectively.

The contents of C14:0, C16:0, C18:2n6c and ΣTFA were significantly higher in G32 than those in G26 (*p* < 0.05, Table 3). The content of C18:1n9t and C22:0 was significantly higher in G29 than in G26 (*p* < 0.05, Table 3). There was no significant difference of other fatty acids among the groups (*p* > 0.05, Table 3).

### 3.3. Effect of Temperature on Ovary and Body Weight at Each Week of Gosling during Brooding

The body weight in G29 was significantly higher than that in G32 and G26 at 2 weeks of age (*p* < 0.05, Table 4). There was no significant difference in body weight at other weeks of age among the groups (*p* > 0.05, Table 4). The ovary weight was significantly higher in G29 than in G32 and G26 at 2, 3 and 4 weeks of age (*p* < 0.05, Table 4). The ovary weight showed no significant difference at 1 week of age among groups (*p* > 0.05, Table 4). The ovary index was significantly higher in G29 than in G32 and G26 at 2, 3 and 4 weeks of age (*p* < 0.05, Table 4). The ovary index showed no significant difference at 1 week of age among groups (*p* > 0.05, Table 4).

### 3.4. Effect of Temperature on Ovarian Development of Gosling at Each Week

**Figure 3:**
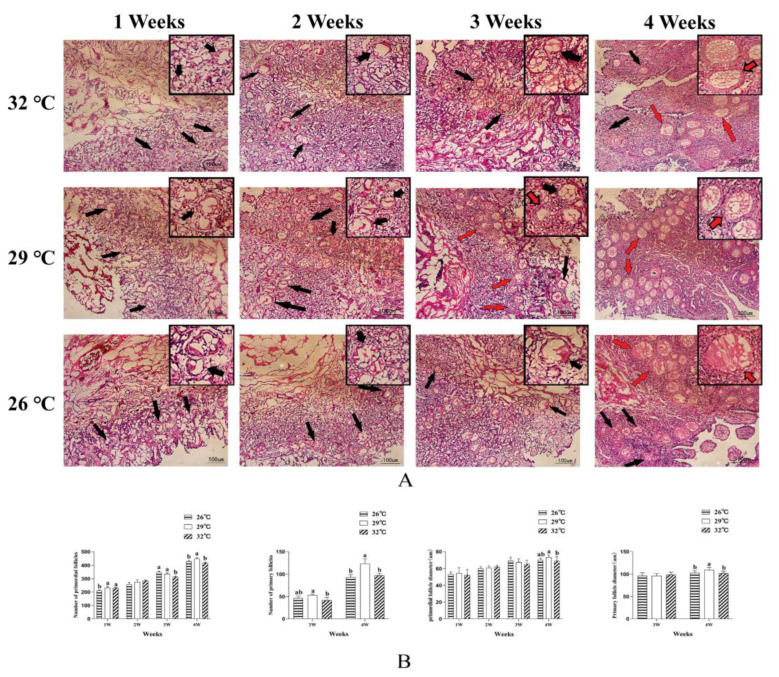
Effect of temperature on follicles’ development of goslings at each week of age. (**A**) HE sections of gosling ovaries at each week of age, a higher magnification is presented in the inserts. Black arrows refer to primordial follicles and red arrows refer to primary follicles. (**B**) Effects of temperature on the number of follicles and diameter of follicles in goslings of different weeks of age. ^a,b^ Different letters in the same stage within an index indicate a significant difference (*p* < 0.05).

During 1–2 weeks, a thin cortex, loosed medulla, and large number of germ cells and follicles accompanied by few primordial follicles were observed. The number of primordial follicles in the ovarian cortex increased at 3 weeks of age, and they were densely distributed in a single layer with a few accompanying primary follicles. The primordial follicles showed multi-layer distribution at 4 weeks of age (Figure 3A). The number of primordial follicles in G32 and G29 was significantly higher than in G26 at 1 week of age (*p* < 0.05, Figure 3B). The number of primordial follicles in G29 was significantly higher than in G32 at 3 and 4 weeks of age (*p* < 0.05, Figure 3B). The diameter of primordial follicles showed no difference among groups in 1 to 3 weeks of age, and was significantly higher in G29 than in G32 at 4 weeks of age (*p* < 0.05, Figure 3B). The number of primary follicles was significantly higher in G29 than in G32 at 3 and 4 weeks of age (*p* < 0.05, Figure 3B). The diameter of primary follicles in G29 was significantly higher than in G32 and G26 at 4 weeks of age (*p* < 0.05, Figure 3B). 

### 3.5. Effects of Temperature on mRNA Expression of Ovarian Development Related Genes in Goslings at Each Week of Age

The expression of *CHK1* was significantly higher in G26 than in G32 and G29 at 1 week of age (*p* < 0.05, Table 5), while the expression of *CHK1* was significantly higher in G29 than in G32 at 2 and 4 weeks of age (*p* < 0.05, Table 5). The expression of *FGF12* was significantly higher in G26 than in G32 and G29 at 1 week of age (*p* < 0.05, Table 5). The expression of *FGF12* was significantly higher in G29 than in G26 and G32 at 3 and 4 weeks of age (*p* < 0.05, Table 5). The expression of *SMAD4* was significantly higher in G29 than in G32 and G26 at 2 and 4 weeks of age, and was significantly higher than that of G26 at 3 weeks of age (*p* < 0.05, Table 5). 

## 4. Discussion

### 4.1. Low Brooding Temperature Facilitates Fatty Acid Absorption in Yolk Sac 

In this study, the weight of the yolk sac showed no significant difference among groups reared at different temperatures during the first 3 days. The energy and protein in the yolk sac of newborn chicks is sufficient to maintain its nutritional needs in the first three days post-hatch [17]. It is suggested that the weight of the yolk sac was not affected by temperature in the first 7 days when the chicks were brooded in 30–32 °C or 24–26 °C [18]. Therefore, the weight of the yolk sac was not affected by temperature.

The lipid of the yolk sac contains major nutrients such as triglycerides, phospholipids with a few cholesterol, and free fatty acids, which serve as substrates for energy metabolism [19]. The fatty acids in the yolk sac are mainly composed of C16 and C18. In this experiment, a lower fatty acid content was observed in chicks with low brooding temperature, which suggests that low temperature could be beneficial to the absorption of fatty acids from the yolk sac during the early days post-hatch. A previous study has shown [20] that the liver of Muscovy ducks could synthesize a large amount of long-chain fatty acids by up-regulating the expression of lipid synthesis genes such as *FAS* during egg laying. It is speculated that the liver of goslings is not fully developed in the early stage, and the body absorbs long-chain fatty acids through the yolk sac to promote the development of organs including the ovary. Therefore, an adequate brooding temperature is crucial so as to maintain the effective absorption of the yolk sac in the first three days.

### 4.2. Appropriate Brooding Temperature Is Conducive to the Body Weight Gain of Goslings

In this study, the body weight of goslings raised at 29 °C was higher than that of birds at 32 °C. In the first weekend of brooding, the body weight of chicks is three-four times that of newborn chicks [21]. Ducklings brooded initially at 28 °C showed a better body weight, weight gain, and feed intake than those brooded of 26 °C or 36 °C [22]. It was observed that high thermal exposure of chicks during the first day post-hatch resulted in body weight loss [23]. Exposure to high temperature during the first 2 days of life caused body weight loss of about 12% in chicks [24], which suggests that a relatively low brooding temperature could be helpful for maintaining body weight. 

In this study, body weight was significantly higher in goslings raised at 29 °C, and a higher body weight was also observed in geese at their 2 weeks of age. Pelicouldo et al. [25] demonstrated that ambient temperature is an important factor affecting the production performance of broilers, and the body weight of chicks incubated at 35 °C was significantly reduced. Kidd et al. [26] showed that every gram of body weight gained at day age was associated with 5 g of body weight gain on the 49th day. Therefore, too high or too low temperatures in the first few days of life would reduce final body weight, and the right temperature is conducive to the body weight gain of goslings. 

### 4.3. Appropriate Brooding Temperature Could Promote Ovary and Follicle Development in Goslings

In this study, the ovarian weight and ovary index were significantly higher in geese reared at 29 °C from 2 to 4 weeks of age. The reproductive performance of Japanese quails was decreased by lowered ovarian weight in high ambient temperature, which was aggravated by liver damage caused by high temperature [27]. Therefore, a suitable temperature is conducive to ovarian development.

Hot environments could increase blood, rectal and uterine temperatures [28], suppress ovarian cyclicity, follicle selection and ovulation [9] and egg laying capability [29]. In this study, the number of primordial follicles and primary follicles, and the diameter of primary follicle were significantly higher in geese raised at 29 °C. Pu et al. [30]. pointed out that the number of follicles decreased with heat stress, and caused marked hyperthermia, reduced ovarian weight and follicle number. High temperature decreased the release of leptin, and then downregulated the growth of ovary follicles and corpus luteum development, promoted ovarian cell apoptosis and inactivated ovarian cell proliferation through the hypothalamo-hypophysial axis [31]. In this study, the diameter of primordial follicles increased much more than that of the primary follicles. Primary follicles were found in the ovaries of ducks 1 week earlier than that of geese. These results indicated that ducks have a more rapid growth rate of ovarian follicles than those of geese during early post-hatch [32], which was consistent with the experimental results of this study. Therefore, appropriate temperature could promote ovarian development, facilitate follicle growth, and increase the activation of primordial follicles. 

### 4.4. Appropriate Brooding Temperature Could Promote the Expression of Genes Related to the Regulation of Ovarian Follicle Development

Phosphorylation of *CDH1* mediated by *CHK1* helps to recognize ubiquitin ligase and promotes effective entry into the S phase of the cell cycle. After silencing *CDK1* and *CHK1* genes [10], ovarian cell proliferation was inhibited and the apoptosis rate was increased. It is reported that *CHK1* was crucial for eliminating the defective nature of oocyte during its meiotic phase [33]. The *CHK1* and *CHK2* dependent DNA damage response controls the number of oocytes [34]. It is speculated that early low temperature brooding is conducive to the rapid absorption of fatty acids in the early stage, and then promotes the rapid activation and differentiation of primordial follicles. Therefore, a higher expression level of *CHK1* was observed in geese reared at 29 °C in this study.

In female reproductive tissues, *FGF12* was highly expressed in the ovary [11]. *FGF12* was highly expressed in geese ovary at 3 and 4 weeks of age when initially reared at 29 °C. It is speculated that appropriate temperature could promote a high level of *FGF12* expression, promote ovarian development, and inhibit premature cell apoptosis. Knockout of *SMAD4* in the ovarian granulosa cells led to the premature luteinization of granulosa cells and eventually premature ovarian failure [35]. In this study, a higher expression of *SMAD4* was observed in geese at 2 and 4 weeks of age when initially reared at 29 °C. It is speculated that the goslings could stimulate the high expression of *SMAD4* and phosphorylate Smad2 or Smad3 to mediate cell proliferation and the cell cycle [36]. 

## 5. Conclusions

Brooding at 29 °C could promote the absorption of fatty acids from the yolk sac. The expression of ovarian development-related genes of *CHK1*, *FGF12*, and *SMAD4*, could participate in promoting early ovarian development and massive activation of primordial follicles in goslings when raised at 29 °C. 

## Figures and Tables

**Table 1:** Primers used for quantitative real-time PCR.

Gene	Accession No.	Forward Primer	Right Primer	Size (bp)
*CHK1*	101790669	GCTGGTGAAGAGGATGACGC	CTCCTGTCCGTGGTGGAGAT	144
*FGF12*	101798106	AGCTCGGATGTTTTCACACC	GCGTCCTTGTTTTTCTCCAA	258
*SMAD4*	101805274	CAGATGCAGCAGCAAGCA	AGCCCTTGACGAAGCTGAG	290
*GAPDH*	101803965	ACTGTCAAGGCTGAGAACGG	AGCTGAGGGAGCTGAGATGA	204

*CHK1*, cell cycle checkpoint kinase 1; *FGF12*, fibroblast growth factor 12; *SMAD4*, Sma-and Mad-related protein 4; *GAPDH*, glyceraldehyde-3-phosphate dehydrogenase.

**Table 2:** Effects of temperature on body weight and yolk sac weight of gosling at various stages.

Item	Time (h)	Temperature		
		G32	G29	G26
Weight (g)	48	107.29 ± 19.18	118.55 ± 13.2	102.93 ± 15.14
	60	117.7 ± 16.47	125.27 ± 26.75	117.72 ± 20.81
	72	138.74 ± 13.25 ^b^	162.12 ± 11.11 ^a^	177.12 ± 3.19 ^a^
Yolk sac weight (g)	48	2.22 ± 0.89	2.80 ± 1.82	1.98 ± 0.72
	60	1.48 ± 0.55	1.14 ± 0.24	1.45 ± 0.27
	72	0.78 ± 0.16	0.62 ± 0.26	0.76 ± 0.13

^a,b^ Different letters indicate significant difference (*p* < 0.05) in a row, the same as below.

**Table 3:** Effects of temperature on fatty acid content of yolk sac fluid.

Item	Temperature		
	G32	G29	G26
C8:0	0.01 ± 0.005	0.01 ± 0.006	0.01 ± 0.004
C14:0	0.12 ± 0.104 ^a^	0.06 ± 0.038 ^ab^	0.05 ± 0.057 ^b^
C16:0	4.91 ± 5.350 ^a^	4.38 ± 2.877 ^ab^	1.47 ± 1.241 ^b^
C18:0	0.75 ± 0.796	1.31 ± 1.213	0.70 ± 0.685
C22:0	1.75 ± 0.893	1.28 ± 1.081	1.12 ± 0.666
ΣSFA	7.83 ± 5.839 ^a^	7.15 ± 4.914 ^a^	3.45 ± 1.929 ^b^
C16:1	2.80 ± 5.037	0.09 ± 0.055	2.15 ± 4.367
C18:1n9t	2.97 ± 1.684 ^ab^	3.38 ± 2.065 ^a^	1.60 ± 0.968 ^b^
C18:1n9c	0.25 ± 0.282	0.24 ± 0.332	0.13 ± 0.143
C18:2n6t	0.08 ± 0.051	0.08 ± 0.093	0.06 ± 0.068
C18:2n6c	0.06 ± 0.049 ^a^	0.04 ± 0.020 ^ab^	0.02 ± 0.018 ^b^
C20:1	0.06 ± 0.023 ^ab^	0.07 ± 0.060 ^a^	0.04 ± 0.034 ^b^
ΣMUFA	5.60 ± 4.052	3.92 ± 2.286	4.04 ± 4.486
C18:3n6	0.10 ± 0.075	0.08 ± 0.038	0.07 ± 0.082
C20:4n6	0.38 ± 0.456	0.24 ± 0.139	0.23 ± 0.148
C20:5n3	0.20 ± 0.232	0.14 ± 0.077	0.34 ± 0.879
ΣPUFA	0.51 ± 0.258	0.39 ± 0.148	0.39 ± 0.230
ΣTFA	15.51 ± 6.643 ^a^	12.73 ± 7.426 ^ab^	8.49 ± 4.902 ^b^

SFA, Saturated fatty acid; MUFA, Monounsaturated fatty acid; PUFA, Polyunsaturated fatty acid; TFA, Total fatty acid.

**Table 4:** Effects of temperature on body weight, ovarian weight and index of goslings at each week of age.

Item	Weeks of Age	Temperature		
		G32	G29	G26
Weight (g)	1	208.96 ± 57.36	192.7 ± 64.48	168.27 ± 27.84
	2	604.23 ± 0.31 ^b^	779.85 ± 26.45 ^a^	607.9 ± 2.80 ^b^
	3	902.5 ± 200.10	1131.45 ± 77.85	879.05 ± 24.45
	4	1678.45 ± 326.15	1678.70 ± 224.90	1704.85 ± 1.25
Ovary weight (g)	1	0.01 ± 0.01	0.03 ± 0.03	0.04 ± 0.02
	2	0.11 ± 0.01 ^b^	0.21 ± 0.03 ^a^	0.1 ± 0.03 ^b^
	3	0.13 ± 0.02 ^c^	0.32 ± 0.01 ^a^	0.21 ± 0.01 ^b^
	4	0.28 ± 0.04 ^b^	0.43 ± 0.08 ^a^	0.24 ± 0.08 ^b^
Ovary index (g/kg)	1	0.07 ± 0.05	0.15 ± 0.14	0.21 ± 0.08
	2	0.17 ± 0.01 ^b^	0.27 ± 0.03 ^a^	0.16 ± 0.04 ^b^
	3	0.15 ± 0.01 ^c^	0.28 ± 0.02 ^a^	0.23 ± 0.01 ^b^
	4	0.17 ± 0.01 ^b^	0.25 ± 0.01 ^a^	0.14 ± 0.05 ^b^

^a–c^ Different letters indicate significant difference in a row (*p* < 0.05). The same as below.

**Table 5:** Effects of temperature on mRNA expression of genes related to ovarian development in goslings at different weeks of age.

Gene	Weeks of Age	Temperature		
		G32	G29	G26
*CHK1*	1	1.65 ± 0.74 ^b^	2.07 ± 0.15 ^b^	5.42 ± 0.44 ^a^
	2	1.28 ± 0.82 ^b^	5.69 ± 3.95 ^a^	4.10 ± 1.64 ^b^
	3	3.54 ± 1.79	2.47 ± 0.72	3.73 ± 0.82
	4	2.08 ± 0.40 ^a^	1.92 ± 0.54 ^a^	1.41 ± 0.08 ^b^
*FGF12*	1	1.88 ± 0.91 ^b^	0.81 ± 0.16 ^b^	18.5 ± 9.35 ^a^
	2	2.62 ± 0.64 ^ab^	4.27 ± 2.17 ^a^	2.31 ± 0.68 ^b^
	3	2.39 ± 0.60 ^b^	16.79 ± 6.25 ^a^	2.62 ± 0.60 ^b^
	4	1.76 ± 0.69 ^b^	6.14 ± 1.37 ^a^	1.34 ± 0.38 ^b^
*SMAD4*	1	1.00 ± 0.18	0.74 ± 0.27	0.70 ± 0.36
	2	2.65 ± 0.65 ^b^	4.53 ± 1.16 ^a^	1.33 ± 0.29 ^c^
	3	3.30 ± 1.78 ^a^	3.13 ± 1.19 ^a^	0.94 ± 0.35 ^b^
	4	0.65 ± 0.34 ^b^	1.12 ± 0.30 ^a^	0.75 ± 0.08 ^b^

## Data Availability

The data presented in this study are available on request from the corresponding author.
